# Biodiversity of Aflatoxigenic *Aspergillus* Species in Dairy Feeds in Bulawayo, Zimbabwe

**DOI:** 10.3389/fmicb.2020.599605

**Published:** 2021-01-21

**Authors:** Nancy Nleya, Lubanza Ngoma, Modupeade C. Adetunji, Mulunda Mwanza

**Affiliations:** ^1^ Department of Animal Health, Northwest University, Mmabatho, South Africa; ^2^ Department of Applied Biology and Biochemistry, National University of Science and Technology, Bulawayo, Zimbabwe; ^3^ Food Security and Food Safety Niche Area, Northwest University, Mmabatho, South Africa; ^4^ Department of Biological Sciences, Trinity University, Lagos, Nigeria

**Keywords:** Aspergillus, biodiversity, feeds, aflatoxins, morphological, molecular

## Abstract

The presence of molds, especially certain species of *Aspergillus*, in food commodities may contribute to aflatoxin contamination. The aim of this study was to determine the biodiversity of *Aspergillus* species in dairy feeds from farms in select locations in Zimbabwe and assess their aflatoxin production potential using a polyphasic approach. A total of 96 feed samples were collected, which consisted of dairy feed concentrate, mixed ration, brewers’ spent grain, and grass from 13 farms during the dry season (August–October, 2016) and the following rainy season (January–March, 2017). A total of 199 presumptive isolates representing four sections from genus *Aspergillus* (*Nigri*, *Fumigati*, *Flavi*, and *Circumdati*) were recovered from the feeds. Section *Flavi*, which includes several aflatoxin producers, constituted 23% (*n* = 46) of the isolates. Species from this section were *A. flavus*, *A. nomius*, *A. oryzae*, *A. parasiticus*, and *A. parvisclerotigenus*, and 39 (84.4%) of these showed evidence of aflatoxin production in plate assays. Of the 46 section *Flavi* isolates examined, some lacked one or more of the five targeted aflatoxin cluster genes (*aflD*, *aflR*, *aflS*, *aflM*, and *aflP*). The presence of the five genes was as follows: *aflD* (76.9%), *aflR* (48.7%), *aflS* (74.4%), *aflM* (64.1%), and *aflP* (79.5%). This study highlights the species diversity of aflatoxigenic fungi that have the potential to contaminate different types of feed for dairy cows. Our findings underscore the importance of preventing contamination of feedstuffs by these fungi so that aflatoxins do not end up in the diets of consumers.

## Introduction

Worldwide mycotoxin contamination of agricultural commodities by members of the *Aspergillus* genus, especially *A. flavus* and *A. parasiticus*, has been reported (Dutta and Das, 2001; Ghiasian and Maghsood, 2011; Ibrahim et al., 2016). These two species, together with *A. nomius*, are well known producers of the mycotoxin, aflatoxin, a secondary metabolite (Rodrigues et al., 2011; Monson et al., 2015). The ability of aflatoxin production by other species from this section such as *A. pseudotamarii*, *A. bombycis*, *A. toxicarius*, *A. parvisclerotigenus*, *A. minisclerotigenes*, and *A. arachidicola* have been documented (Ito et al., 2001; Pildain et al., 2008; Matumba et al., 2015). Aflatoxin contamination of food and feeds has gained global attention due to their negative effects on the health of both humans and animals (Fink-Grernmels, 1999; Zain, 2011; Arapcheska et al., 2015; Kumar et al., 2017). The more serious mycotoxins produced by *Aspergillus* species include aflatoxin (AF) B_1_, B_2_, G_1_, and G_2_ (Kumar et al., 2017). AFB_1_ is the most potent of them all and has been implicated as a causative agent of human hepatocarcinoma (Liu and Wu, 2010; Baranyi et al., 2013; Patel et al., 2015). Biosynthesis of aflatoxins involves participation of several enzymes and regulatory proteins whose genes are found in a single cluster of 70–75 kb in size. The order of these genes in *Aspergillus* is highly conserved (Woloshuk and Prieto, 1998; Yu et al., 2004; Adhikari et al., 2016). Not all section *Flavi* species produce aflatoxins (Atanda et al., 2006, 2011; Degola et al., 2007; Yazdani et al., 2010). The inability to produce aflatoxins by some species in this section is mainly due to mutations in at least one of the genes in the aflatoxin biosynthetic gene cluster (Degola et al., 2007; Adhikari et al., 2016).

Accurate identification of members of the *Aspergillus* genus using a single method is difficult due to their morphological similarities (Moslem et al., 2010; Zulkifli and Zakaria, 2017). Also, use of macro- and micro-morphological characters alone for identification is tedious and time consuming (Henry et al., 2000). To accelerate the species identification process, molecular markers such as the internal transcribed spacer (ITS) region have become the primary locus for fungal identification (Schoch et al., 2012; Raja et al., 2017). Extrolite profiling (e.g., types of aflatoxin produced) has also been used to aid in the identification and classification of Aspergilli though less frequently (Frisvad et al., 2007). Authors have proposed several media for cultural identification of aflatoxin producers (Bothast and Fennell, 1974; Lin and Dianese, 1976; Davis et al., 1987; Atanda et al., 2006, 2011). These media have chemical additives that enhance aflatoxin production for easy detection under UV radiation at 365 nm (Fente et al., 2001; Midorikawa et al., 2008). However, there are also limitations with using these types of plate assays (Suzuki and Iwahashi, 2016). Researchers report circumventing culture-based limitations by amplifying genes coding for key enzymes in the aflatoxin biosynthetic pathway (*aflD*, *aflR*, *aflS*, *aflM*, *aflP*) for identification of aflatoxigenic species (OBrian et al., 2007; Abdel-Hadi et al., 2011a; Mejía-Teniente et al., 2011; Baranyi et al., 2013; Zhi et al., 2013; Verheecke et al., 2015). Thus, for precise identification of aflatoxigenic *Aspergillus* isolates, a polyphasic approach is encouraged (Adetunji et al., 2019).

Animal feeds are formulated using agricultural commodities such as cereal grains and oil seed cakes. Cereal grain stovers and grass as silage can also be used as animal feed (Pleadin, 2015; Morrison et al., 2017). Moreover, the high cost of feed has led to the addition of stale bread, kitchen, and bakery wastes to extend its use, while the scarcity of protein sources for animal feeds has led to amendments with items such as brewers’ spent grain (BSG; Li et al., 2014). These waste products are usually tainted with fungi which may be a contributing factor in mycotoxin contamination in cattle feed. Upon ingestion by the cow, AFB_1_ is biotransformed into its hydroxylated metabolite aflatoxin M_1_ (AFM_1_), which is secreted in the milk (Britzi et al., 2013; Giovati et al., 2015) and becomes a route through which aflatoxins can be introduced into humans. Therefore, it is important to assess the quality of the feed fed to dairy cows in order to curb the transfer of the aflatoxins into milk. In this study, the diversity of *Aspergillus* species in dairy feeds, as well as their potential contamination with aflatoxins, was assessed using cultural-based and molecular methods.

## Materials and Methods

### Sample Collection

A total of 96 feed samples consisting of dairy feed concentrate (CN, *n* = 32), mixed ration (MR, *n* = 35), brewers’ spent grain (BSG, *n* = 7), and grass (GR, *n* = 22) were collected from 13 farms during the dry season (August–October, 2016) and the following rainy season (January–March, 2017). Samples were collected in sterile polythene zip-lock bags, which were transported in cooler boxes to the laboratory where they were ground to a fine powder using an IKA® M20 universal batch mill (Germany) and stored in the freezer at −20°C until time for analysis.

### Isolation and Presumptive Identification of *Aspergillus* Fungi

Fungal isolation from feed samples followed the method of Aliyu et al. (2012) with some modifications as follows: feed samples weighing 1 g were suspended in 9 ml of sterile distilled water and mixed through vortexing. The sample was allowed to settle and 1 ml of the supernatant transferred into a test tube containing 9 ml of distilled water. The 10-fold serial dilution was carried out up to 10^−6^. This was followed by spread plating 0.1 ml of each dilution onto potato dextrose agar (PDA) supplemented with chloramphenicol (100 μg/ml). The plates were incubated at 25 ± 2°C for 3–7 days. Pure cultures were obtained through the single spore method (Almoammar et al., 2013). Since closely related *Aspergillus* species within each section have similar colony colors, we used this macro-morphological phenotype to identify our presumptive *Aspergillus* isolates to section level (Almoammar et al., 2013; Mostafa and Amer, 2013).

### Screening for Aflatoxin-Producing Isolates

Each presumptive *Aspergillus* isolate was screened for the production of aflatoxin using multiple plating methods. Yeast Extract Sucrose (YES) agar (2% yeast extract, 15% sucrose, and 1.5% agar) shows the presence of aflatoxin through an induced color change plum red when exposed to ammonium hydroxide vapors (Yazdani et al., 2010). Aflatoxin-producing Aspergilli grown on Neutral Red Desiccated Coconut Agar (NRDCA; 20% desiccated coconut, 2% agar, and 0.03% neutral red stain; Atanda et al., 2011) and Neutral Red Desiccated Coconut Agar with β-cyclodextrin (β-CNRDCA; 20% desiccated coconut, 2% agar, 0.03% neutral red stain, and 0.03% β-cyclodextrin; Adetunji et al., 2019) will display a yellow/orange ring around the growing colony that will fluoresce on the reverse side of the plate when exposed to long-wave UV light (365 nm).

### Molecular Identification of Isolates

For molecular investigations, we first inoculated each presumptive isolate on PDA and incubated them at 28°C for 72 h. DNA extraction utilized a Zymo Research Quick-DNA™ Fungal/Bacterial Miniprep Kit (The Epigenetics Company™, United States), and purity was assessed using a Nano-200 Microspectrometer (Allsheng Instruments Co., Ltd). DNA was stored at −20°C until time for use (Egbuta et al., 2015).

To refine our presumptive isolate identifications to species level, we amplified the internal transcribed spacer (ITS) region of each isolate’s DNA (ITS1-5.8S-ITS2) following the methods of Egbuta et al. (2015) with some modifications. Briefly, universal primers, ITS1 (5'-CTTGGT CAT TTA GAG GAA GTA A-3') and ITS4 (5'-TCC TCC GCT TAT TGA TATGC-3') synthesized by Inqaba Biotec (Pretoria, South Africa) were used. The PCR reaction consisted of 12.5 μl of 2× PCR master mix, 0.5 μl of each 25 μM primer, and 5 μl of DNA and constituted to a final volume of 25 μl with nuclease-free water and was carried out in a T100™ thermal cycler (Bio-Rad, Singapore) under the following conditions as follows: pre-dwelling at 95°C for 3 min, 35 cycles of denaturation at 95°C for 1 min, annealing at 58°C for 45 s, extension at 72°C for 1 min 30 s, post-dwelling at 72°C for 10 min, and holding at 4°C until samples were retrieved (Egbuta et al., 2015).

PCR amplification of key regulatory and structural genes from the aflatoxin pathway (*aflD*, *aflR*, *aflS*, *aflM*, *aflP*) involved primer pairs and cycling parameters previously described in other studies (Table 1). All PCR products were electrophoresed on a 1% agarose gel and visualized using a Bio-Rad Molecular Image® Gel Doc™ XR+ with Image Lab™ software (Bio-Rad, CA, United States). The amplicons were sent to Inqaba Biotec (South Africa) for sequencing. Analysis and cleaning of chromatograms were done using Finch TV software version 1.4.0 (Frickmann et al., 2015). Our *Aspergillus* sequences were queried against previously accessioned sequences in GenBank (National Centre for Biotechnology and Information) using the Basic Local Alignment Search Tool (BLAST; Magnani et al., 2005; Castrillo et al., 2012; Zarrin and Erfaninejad, 2016). Species identification was based on the best score (≥99% similarity; Gajjar et al., 2013; Frickmann et al., 2015; Rossi-Tamisier et al., 2015; Beye et al., 2017).

**Table 1:** Primer sequences and PCR conditions used for the detection of aflatoxigenic *Aspergillus* isolated from dairy feeds.

Prime pair	Target gene	Primer sequence (5' → 3')	PCR conditions							Reference
			1	2	3	4	5	6	Product size (bp)	
Nor-1F Nor-1R	*aflD*	ACCGCTACGCCGGCACTCTCGGCAC GTTGGCCGCCAGCTTCGACACTCCG	94°C:10 min	94°C:1 min	65°C:1 min	72°C:2 min	33	72°C:min s	400	
Ver-1F Ver-1R	*aflM*	GCCGCAGGCCGCGGAGAAAGTGGT GGGGATATACTCCCGCGACACAGCC	95°C:4 min	95°C:1 min	58°C:1 min	72°C:30s	30	72°C:10 min	600	Rashid et al., 2008; Abd El-Aziz et al., 2015
Omt-1F Omt-1R	*aflP*	GTGGACGGACCTAGTCCGACATCAC GTCGGCGCCACGCACTGGGTTGGGG	94°C:5 min	94°C:1 min	75°C:2 min	72°C:2 min	33	72°C:10 min	797	
aflR-1F	*aflR*	TATCTCCCCCCGGGCATCTCCCGG GTCGGCGCCACGCACTGGGTTGGGG	95°C:4 min	94°C:1 min	60°C:1 min	72°C:30s	30	72°C:10 min	1,000	
aflR-1R										
AflS-1F AflS-2R	*aflS*	TGAATCCGTACCCTTTGAGG GGAATGGGATGGAGATGAGA	95°C:10 min	95°C:50s	58°C:50s	72°C:2 min	30	72°C:5 min	684	Gallo et al., 2012; Fakruddin et al., 2015

1, Pre-dwelling; 2, Denaturation; 3, Annealing; 4, Extension; 5, Cycles; 6, Post-dwelling.

## Results

### Identification and Characterization of Isolates

**Figure 1:**
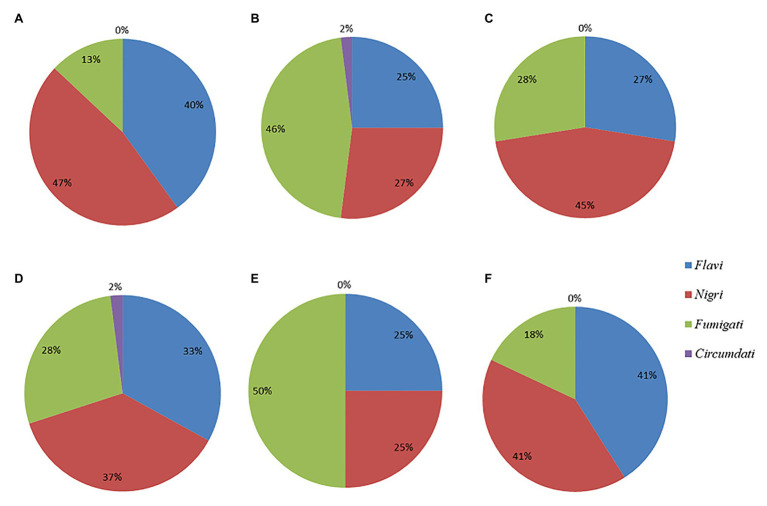
Pie charts showing the percent distributions of the four *Aspergillus* sections represented by the **(A)** dry season isolates **(B)** rainy season isolates **(C)** CN isolates **(D)** MR isolates **(E)** BSG isolates, and **(F)** GR isolates.

A sample population of 123 dry-season fungal isolates and 78 rainy-season fungal isolates were presumptively identified as *Aspergillus* and segregated by colony color into two main subgenera, namely, *Circumdati* and *Fumigati*, and four sections: *Fumigati* (blue colonies), *Flavi* (green colonies), *Circumdati* (yellow colonies), and *Nigri* (black colonies). The relative abundances of isolates from each section during the dry season and rainy season, as well as in the different feeds, are shown in Figure 1.

The ITS PCR amplicons were of the expected size for the region (600 bp). A total of 107 dry season and 78 rainy season sequences were of good enough quality to BLAST query. The remaining amplicons (*n* = 14 isolates) yielded poor sequences; therefore, these were not accessioned or subjected to further analysis. BLAST query of the ITS sequences of 185 presumptive *Aspergillus* isolates revealed that 83 of the isolates from the dry season had 99–100% similarity to the following species: *A. niger*, *A. awamori*, *A. tubingensis*, *A. flavus*, *A. nomius*, *A. oryzae*, *A. parasiticus*, *A. fumigatus*, *A. foetidius*, *A. chavalieri*, *A. sydowii*, *A. brasiliensis*, and *A. cristatum*. The remaining 24 belonged to other genera such as *Byssochlamys*, *Cladosporium*, *Penicillium*, *Talaromyces*, *Eurotium*, and *Didymella*. From the rainy season, 59 showed the same percentage similarity to *A. niger*, *A. awamori*, *A. tubingensis*, *A. flavus*, *A. nomius*, *A. oryzae*, *A. fumigatus*, *A. ochraceus*, *A. welwitschiae*, *A. phoenicis*, *A. parvisclerotigenus*, and *A. japonicus*. The other 17 isolates belonged to *Byssochlamys*, *Cladosporium*, *Penicillium*, *Talaromyces*, *Alternaria*, *Paecilomyces*, *Rhizopus*, and *Sarocladium*. The isolates were accessioned to GenBank and given numbers MG659595 to MG659694 for dry season isolates and MH270529 to MH270615 for rainy season isolates (Supplementary Table S1).

Out of the 142 isolates identified as members of the *Aspergillus* genus, 48 belonged to section *Flavi*. The distribution of the species in the various feeds is shown in Table 2. *A. flavus* (section *Flavi*) had the highest number of isolates in the dry season and were mainly from grass, whereas *A. fumigatus* (section *Fumigati*) was more predominant in the rainy season being isolated most frequently from the mixed ration samples. Section *Nigri* was dominated by *A. niger*, and most of these isolates were from the mixed ration and grass feeds. Only one isolate of *A. ochraceus* represented section *Circumdati* and it was isolated from a rainy season mixed ration feed sample (Table 2).

**Table 2:** Distribution of species from various sections in the different feeds.

Section	Species	Number of isolates					
		Dry season	Rainy season	MR	CN	GR	BSG
*Flavi*	*A. flavus*	26	8	11	6	14	3
	*A. oryzae*	4	5	4	2	2	0
	*A. parasiticus*	2	0	2	0	0	0
	*A. nomius*	1	1	0	0	2	0
	*A. parvisclerotigenus*	0	1	1	0	0	0
*Nigri*	*A. niger*	24	7	12	6	12	1
	*A. tubingensis*	6	1	0	4	3	0
	*A. awamori*	4	1	3	0	1	1
	*A. chevalieri*	1	1	2	0	0	0
	*A. cristatus*	1	0	1	0	0	0
	*A. foetidus*	1	0	0	0	1	0
	*A. sydowii*	1	0	0	0	0	1
	*A. brasiliensis*	1	0	0	0	1	0
	*A. phoenicis*	0	4	2	2	0	0
	*A. japonicus*	0	1	1	0	0	0
	*A. welwitschiae*	0	1	0	1	0	0
*Fumigati*	*A. fumigatus*	11	27	16	8	8	6
*Circumdati*	*A. ochraceus*	0	1	1	0	0	0

MR, mixed ration; CN, concentrate; GR, grass; BSG, brewers’ spent grain.

### Findings Relating to Aflatoxin Production

**Figure 2:**
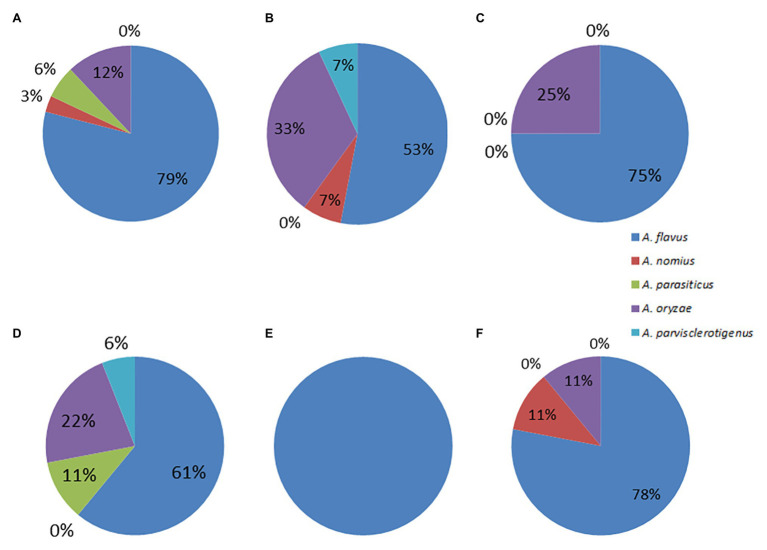
Pie charts showing the percent distributions of species within section *Flavi* represented by the **(A)** dry season isolates **(B)** rainy season isolates **(C)** CN isolates **(D)** MR isolates **(E)** BSG isolates, and **(F)** GR isolates.

**Figure 3:**
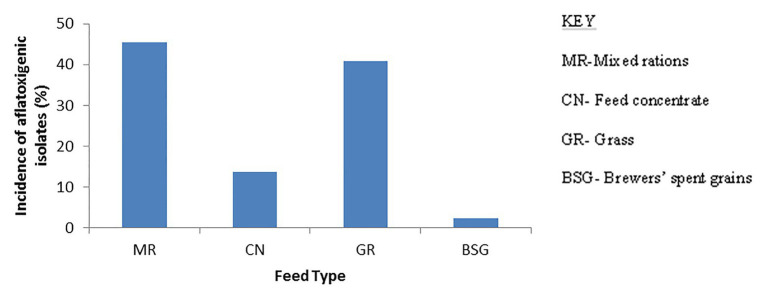
Bar charts showing occurrence of aflatoxigenic *Aspergillus* strains in the different types of feeds. The mixed ration harbored most of the aflatoxigenic strains and BSG the least.

Assessment of aflatoxin-producing ability was carried out on all the isolates belonging to the *Aspergillus* genus. Results for plate assays are shown in Table 3. Out of the 142 isolates, 42 tested positive for aflatoxin on YES agar, 27 from the dry season, and 15 from the rainy season (Supplementary Table S2). From section *Flavi*, *A. flavus* dominated for both seasons. Other members of the same section included *A. oryzae*, *A. parasiticus*, and *A. nomius*. Some isolates outside section *Flavi*, such as *A. tubingensis* ND2 and ND9, *A. ochraceus* NR2, *A. fumigatus* NR37, and *A. japonicus* NR67 also tested positive on YES. For NRDCA and β-CNRDCA, some isolates only showed the yellow ring with no fluorescence under UV light, others had no yellow ring but showed some fluorescence, and others showed both a yellow ring and the fluorescence in the reverse (Supplementary Table S2). Only isolates showing both yellow ring and fluorescence were considered as positive for aflatoxin production. Distribution of the aflatoxin biosynthesis cluster genes across the 142 *Aspergillus* isolates analyzed is given in Table 4. The percentage occurrences of the aflatoxin biosynthetic genes were as follows: (51%) *aflD* (14%) *aflR* (29%) *aflS* (14%) *aflM*, and (32%) *aflP*. Most of the isolates harbored at least one of the genes, and eight possessed all the five genes analyzed (Supplementary Table S3). Confirmation of aflatoxin-producing isolates using combined methods identified 30 positive isolates all belonging to section *Flavi*, namely, *A. flavus*, *A. oryzae*, *A. parasiticus*, *A. nomius*, and *A. parvisclerotigenus* (Table 5). The majority of the isolates were from the dry season and isolated from mixed ration and grass (Figure 2) and the least from brewers’ spent grain (Figure 3).

**Table 3:** Plate assay results for aflatoxin producing ability of *Aspergillus* isolates.

Media	YES	NRDCA	β-CNRDCA
Dry season	28	21	22
Rainy season	14	8	7
Total	42	9	29

**Table 4:** Presence and absence of five aflatoxin cluster genes in *Aspergillus* isolates.

	*aflD*		*aflR*		*aflS*		*aflM*		*aflP*	
	Positive	Negative	Positive	Negative	Positive	Negative	Positive	Negative	Positive	Negative
Dry season	37	46	13	70	26	57	12	71	21	62
Rainy season	37	22	7	52	15	44	8	51	25	34
Total	74	68	20	122	41	101	20	122	46	96
Percentage (%)	**52**	**48**	**14**	**86**	**29**	**71**	**14**	**86**	**32**	**68**

**Table 5:** Aflatoxigenic isolates based on polyphasic examination.

Isolate vouche	Season	Feed type	Species	GenBank accession #
ND26	Dry	MR	*A. flavus*	MG659620
ND27	Dry	GR	*A. nomius*	MG659621
ND29	Dry	CN	*A. oryzae*	MG659623
ND30	Dry	GR	*A. flavus*	MG659624
ND31	Dry	MR	*A. flavus*	MG659625
ND32	Dry	MR	*A. parasiticus*	MG659626
ND33	Dry	GR	*A. flavus*	MG659627
ND34	Dry	GR	*A. flavus*	MG659628
ND35	Dry	GR	*A. oryzae*	MG659629
ND37	Dry	MR	*A. flavus*	MG659631
ND38	Dry	GR	*A. flavus*	MG659632
ND39	Dry	MR	*A. oryzae*	MG659633
ND40	Dry	CN	*A. flavus*	MG659634
ND41	Dry	GR	*A. flavus*	MG659635
ND51	Dry	GR	*A. flavus*	MG659645
ND52	Dry	GR	*A. flavus*	MG659646
ND59	Dry	MR	*A. flavus*	MG659653
ND75	Dry	MR	*A. flavus*	MG659669
ND93	Dry	MR	*A. parasiticus*	MG659687
ND96	Dry	GR	*A. oryzae*	MG659690
ND99	Dry	GR	*A. flavus*	MH270605
NR3	Rainy	MR	*A. flavus*	MH270531
NR20	Rainy	CN	*A. flavus*	MH270548
NR31	Rainy	BSG	*A. flavus*	MH270559
NR35	Rainy	MR	*A. oryzae*	MH270563
NR40	Rainy	MR	*A. parvisclerotigenus*	MH270568
NR46	Rainy	MR	*A. flavus*	MH270574
NR50	Rainy	MR	*A. flavus*	MH270578
NR66	Rainy	MR	*A. oryzae*	MH270594
NR72	Rainy	GR	*A. nomius*	MH270600

MR, mixed ration; CN, concentrate; GR, grass; BSG, brewers’ spent grain.

## Discussion

It has been reported that ingredients used in animal feed formulations can be contaminated by molds that are capable of producing aflatoxins (Gelven, 2010). This has led to extensive research in order to understand the diversity of *Aspergillus* species in feeds and feedstuffs (Variane et al., 2018). Findings from previous studies have indicated the presence of a wide range of *Aspergillus* species in feeds and feedstuffs. However, in Zimbabwe no work has reported on *Aspergillus* diversity and levels of aflatoxin contamination in animal feed.

Our findings showed that most of the isolates from our feed samples belonged to subgenera *Fumigati* (section *Fumigati*) and *Circumdati* (sections *Nigri*, *Circumdati*, and *Flavi*) based on observed morphological characteristics. The characteristic feature used in the identification of the isolates was the colony color that classified the isolates as follows: section *Fumigati* (blue), *Nigri* (black), *Circumdati* (yellow), and *Flavi* (green). However, use of only macro-morphological features has limitations since it does not guarantee accurate identification to species level. In order to identify the species with greater confidence, colony color should be augmented by examination of micro-morphological features of the isolates such as conidiophore, vesicle, metulae, phialides, and conidia, which was not done in our case. Alteratively, molecular analyses were performed on the isolates in order to resolve the limitations of the cultural methods. ITS sequence analysis using the BLAST search tool identified the black isolates as *A. niger*, *A. brasiliensis*, *A. tubigensis*, *A. foetidus*, *A. phoenicis*, *A. welwitschiae*, *A. awamori*, *A. cristatus*, *A. sydowii*, *A. chevaliieri*, and *A. japonicus*. The green isolates were identified as *A. flavus*, *A. oryzae*, *A. nomius*, *A. parasiticus*, and *A. parvisclerotigenus*. This shows the limitation of using morphological features for characterization; besides the process being time consuming and laborious (Henry et al., 2000) it can lead to misidentification. Therefore, there is a need for a polyphasic approach for the proper identification of the isolates (Adetunji et al., 2019). This work is limited in the sense that only the ITS region of the *Aspergillus* isolates was amplified. The use of ITS alone for the identification of some fungal genera has received criticism, especially in the discrimination of closely related species for genera such as *Aspergillus*, *Cladosporium*, *Fusarium*, *Penicillium*, and *Trichoderma* (Raja et al., 2017). The inability of the ITS region to differentiate between identical species such as *A. flavus* and *A. oryzae* has also been highlighted by Adetunji et al. (2019, 2020). Since amplification of ITS alone is not sufficient, further analysis of the isolates using other gene markers such as calmodulin (*caM*) and β-tubulin (*benA*) is therefore recommended in order to improve species identifications.

Some *Aspergillus* species outside section *Flavi*, such as those from sections *Nidulantes* and *Ochraceorosei*, have been reported to have the ability to produce aflatoxins (Cary et al., 2005; Varga et al., 2009); therefore, all 142 isolates were screened for aflatoxin producing potential (Supplementary Table S3). Sudini et al. (2015) indicated chances of having false-positive and false-negative results when using cultural methods as some non-aflatoxigenic species can produce yellow pigmentation and also fluoresce under UV light. This has been previously highlighted by Stark (2009) and Atanda et al. (2011). In this study, one *A. fumigatus* isolate NR12 exhibited some fluorescence and presence of yellow ring on β-CNRDCA, whereas for YES and molecular analysis the results were negative for the same isolate for aflatoxin production. Identification of these isolates based on the use of plate assays classified them as aflatoxin producers (Supplementary Table S2) while they are non-aflatoxigenic strains. Similarly, *A. ochraceus* isolate NR2 tested positive in both plate assays but lacked all aflatoxin cluster genes examined. This species is known for ochratoxin A production, which is able to fluoresce under UV light (Mohamed et al., 2013; Bueno et al., 2017); hence, using cultural methods would have classified it inappropriately as an aflatoxin producer. Similar observations were observed for isolates *A. oryzae* NR57 and *A. fumigatus* NR37, which indicated aflatoxin-positive results for both plate assays yet lacked one or more of the genes examined during molecular analysis. On the other hand, classification of isolates *A. flavus* NR3 and *A. oryzae* NR35 using only the coconut-based media suggested both as non-aflatoxigenic strains, yet the YES plate assay suggested they were aflatoxin positive, and both contained all the genes examined. Thus, it is important to classify organisms using combined methods. Another isolate, *A. parvislerotigenus* NR40, gave negative results for plate assays but had all major aflatoxin cluster genes analyzed in this study. It could have been that the isolate produced the toxins in low enough quantities to escape detection by culture assays. Therefore, in addition to the plate assays, the presence of aflatoxin could have been confirmed by chromatographic methods such as thin layer chromatography, high performance liquid chromatography (HPLC) or liquid chromatography with tandem mass spectrometry (LC-MS/MS).

Use of enhancing agents like β-cyclodextrin to improve fluorescence of aflatoxins has been reported (Abbas et al., 2004; Stark, 2009; Degola et al., 2012). In this study, only one isolate, *A. japonicus* NR67, had enhanced fluorescence on β-cyclodextrin-supplemented NRDCA. This may suggest that the use of plain media or supplemented media does not affect the results. Although this isolate gave positive results on YES and exhibited some fluorescence under UV light on β-cyclodextrin-supplemented media, it was classified as a non-aflatoxigenic strain as none of the aflatoxin cluster genes were amplified. There are no reports of aflatoxin production by species from sections *Nigri* and *Fumigati*, which is also in agreement with our observation from this study. The fluorescence under UV light exhibited by the isolates suggests possibility of production of other metabolites that can fluoresce. Investigations into the identities of these extrolites need to be carried out in the future.

Characterization of the isolates using molecular methods showed the presence of the *aflD* gene in 52% of the isolates. According to previous studies (Cleveland et al., 2009; Abdel-Hadi et al., 2011a,b; Yu, 2012), *aflD* codes for an enzyme that converts norsolorinic acid, the first stable intermediate in the aflatoxin biosynthetic pathway, to averantin. The other two structural genes, *aflM* and *aflP*, had a frequency of 14 and 25%, respectively. The aflatoxin biosynthetic pathway is said to be regulated by the *aflR* gene which encodes for transcriptional activators for the structural genes, however the discovery of yet another gene, *aflJ*, showed that *aflR* does not solely regulate the biosynthesis of aflatoxins since its activities were affected by *aflS* (Cleveland et al., 2009). Therefore, in this study, we used *aflD*, *aflR*, and *aflS* as the key markers for discriminating aflatoxigenic species from non-aflatoxigenic strains. In this study, detection of *aflR* was low with only 17% of the isolates showing the presence of the gene. This is in agreement with Abdel-Hadi et al. (2011b) who highlighted failure of *aflR* in discriminating aflatoxin producers from non-aflatoxin producing isolates. Similarly, Adetunji et al. (2019) observed the presence of the *aflR* gene in some *A. oryzae* isolates from cashew nuts even though these isolates tested negative for aflatoxin production. A much higher percentage of the isolates (23%) showed the presence of *aflS*. This suggests that *aflS* may be the main regulatory gene in the isolates found in Zimbabwe. The inability of amplification by some of the genes in the isolates could be due to the fact that they could not be expressed under the lab conditions and there is a need to provide conditions that can lead to the expression of the genes.

Isolates from section *Flavi* had an occurrence frequency of 34% across the feeds (Supplementary Table S4). This is a cause of concern as this section is well known for its aflatoxin producing species, *A. flavus* (Rodrigues et al., 2007). From this study, 88% of the isolates from section *Flavi* tested positive for aflatoxin production. *A. flavus* constituted 71% of the isolates in this section, and 56% of these isolates showed the potential to produce aflatoxin using both the cultural and molecular methods of validation. *Aspergillus oryzae* has not been associated with aflatoxin production and has been used in the production of fermented foods (Chang et al., 2006); however, six out of the nine isolates identified as *A. oryzae* exhibited aflatoxin production potential in our plate assays. Reconfirmation of the identities of these six isolates should involve BLAST query of other conserved genomic loci such as calmodulin (*caM*) and β-tubulin (*benA*). *Aspergillus oryzae* is the domesticated form of *A. flavus*, and the duo are morphologically identical (Payne et al., 2006); hence, there is a possibility that the accessioned sequences in GenBank were misidentified by their submitters. However, the presence of aflatoxin regulatory gene, *aflR* has been reported in *A. oryzae* (Kusumoto et al., 1998; Lee et al., 2006; Adetunji et al., 2019), but they were found to be non-aflatoxin producers. The presence of *aflR* cannot be readily associated with aflatoxin production, since any of 25 genes in the aflatoxin pathway could be present and non-functional.

This study reports for the first time the biodiversity of aflatoxigenic *Aspergillus* species in feeds used in dairy farming in Zimbabwe, and the findings showed the presence of four sections of *Aspergilli* with the predominating section being *Nigri*. *Nigri* species are non-aflatoxigenic; however, some are capable of producing ochratoxin A, another regulated mycotoxin. This wide diversity could be attributed to use of contaminated ingredients. Omeiza et al. (2018) highlighted strain diversity as a risk associated with unregulated trade routes used by farmers in Africa when acquiring ingredients for animal feeds. Aflatoxin-producing fungi have a preference for oil seed crops such as maize, cotton, and peanut. The most contaminated feed type was mixed ration, which is usually made by mixing mainly waste products of oil extraction from seeds and other ingredients. Cottonseed cake was the main waste product used by most farmers for the mixed ration. Therefore, farmers are encouraged to avoid contaminated ingredients for formulating mixed rations. The fact that feed producers could be using potentially contaminated ingredients is a serious issue. The local government should regulate these cottonseed meal producers and force them to assess the presence of aflatoxin in their product before they can sell it to growers.

## Data Availability Statement

The datasets presented in this study can be found in online repositories. The names of the repository/repositories and accession number(s) can be found below: https://www.ncbi.nlm.nih.gov/, MG659595 to MG659694 https://www.ncbi.nlm.nih.gov/, MH270529 to MH270615.

## Author Contributions

NN did the isolation and characterization of the isolates from the samples. MA assisted with the assaying for aflatoxin production by the isolates, reviewing and proof reading of the article. LN assisted with the molecular characterization. MM helped with the supervision of the whole research. All authors contributed to the article and approved the submitted version.

### Conflict of Interest

The authors declare that the research was conducted in the absence of any commercial or financial relationships that could be construed as a potential conflict of interest.
