# Supplementary material for: Biodiversity of Aflatoxigenic Aspergillus Species in Dairy Feeds in Bulawayo, Zimbabwe
Source: Front Microbiol. 2021 Jan 21;11:599605. doi: 10.3389/fmicb.2020.599605 (PMC7859627; doi:10.3389/fmicb.2020.599605)
Supplement: Supplementary file 1 [file Table_1.docx]

**Table S1**. ITS Accession numbers

| **Isolate voucher** | **Species** | **Season** | **Feed Type** | **GenBank Accession #** |
| --- | --- | --- | --- | --- |
| ND1 | *A. niger* | Dry | Grass | MG659595 |
| ND2 | *A. tubingensis* | Dry | Concentrate | MG659596 |
| ND3 | *A. awamori* | Dry | Mixed Ration | MG659597 |
| ND4 | *A. niger* | Dry | Mixed ration | MG659598 |
| ND5 | *A. tubingensis* | Dry | Grass | MG659599 |
| ND6 | *A. awamori* | Dry | Mixed Ration | MG659600 |
| ND7 | *A. niger* | Dry | Grass | MG659601 |
| ND8 | *A. tubingensis* | Dry | Concentrate | MG659602 |
| ND9 | *A. tubingensis* | Dry | Grass | MG659603 |
| ND10 | *A. niger* | Dry | Concentrate | MG659604 |
| ND11 | *A. niger* | Dry | Grass | MG659605 |
| ND12 | *A. niger* | Dry | Grass | MG659606 |
| ND13 | *A. niger* | Dry | Grass | MG659607 |
| ND20 | *A. fumigatus* | Dry | Brewers’ Spent Grain | MG659614 |
| ND21 | *A. fumigatus* | Dry | Grass | MG659615 |
| ND25 | *A. flavus* | Dry | Grass | MG659619 |
| ND26 | *A. flavus* | Dry | Mixed Ration | MG659620 |
| ND27 | *A. nomius* | Dry | Grass | MG659621 |
| ND28 | *A. flavus* | Dry | Grass | MG659622 |
| ND29 | *A. oryzae* | Dry | Concentrate | MG659623 |
| ND30 | *A. flavus* | Dry | Grass | MG659624 |
| ND31 | *A. flavus* | Dry | Mixed Ration | MG659625 |
| ND32 | *A. parasiticus* | Dry | Mixed Ration | MG659626 |
| ND33 | *A. flavus* | Dry | Grass | MG659627 |
| ND34 | *A. flavus* | Dry | Grass | MG659628 |
| ND35 | *A. oryzae* | Dry | Grass | MG659629 |
| ND36 | *A. flavus* | Dry | Mixed Ration | MG659630 |
| ND37 | *A. flavus* | Dry | Mixed Ration | MG659631 |
| ND38 | *A. flavus* | Dry | Grass | MG659632 |
| ND39 | *A. oryzae* | Dry | Mixed Ration | MG659633 |
| ND40 | *A. flavus* | Dry | Concentrate | MG659634 |
| ND41 | *A. flavus* | Dry | Grass | MG659635 |
| ND44 | *A. niger* | Dry | Concentrate | MG659638 |
| ND45 | *A. cristatus* | Dry | Mixed Ration | MG659639 |
| ND51 | *A. flavus* | Dry | Grass | MG659645 |
| ND52 | *A. flavus* | Dry | Grass | MG659646 |
| ND54 | *A. awamori* | Dry | Grass | MG659648 |
| ND55 | *A. niger* | Dry | Mixed Ration | MG659649 |
| ND56 | *A. niger* | Dry | Concentrate | MG659650 |
| ND57 | *A. fumigatus* | Dry | Concentrate | MG659651 |
| ND58 | *A. niger* | Dry | Mixed Ration | MG659652 |
| ND59 | *A. flavus* | Dry | Mixed Ration | MG659653 |
| ND60 | *A. awamori* | Dry | Brewers’ Spent Grain | MG659654 |
| ND61 | *A. fumigatus* | Dry | Mixed Ration | MG659655 |
| ND62 | *A. niger* | Dry | Concentrate | MG659656 |
| ND63 | *A. flavus* | Dry | Brewers’ Spent Grain | MG659657 |
| ND64 | *A. niger* | Dry | Grass | MG659658 |
| ND65 | *A. niger* | Dry | Concentrate | MG659659 |
| ND67 | *A. niger* | Dry | Grass | MG659661 |
| ND68 | *A. niger* | Dry | Grass | MG659662 |
| ND69 | *A. fumigatus* | Dry | Grass | MG659663 |
| ND71 | *A. niger* | Dry | Grass | MG659665 |
| ND73 | *A. fumigatus* | Dry | Brewrers’ Spent Grain | MG659667 |
| ND74 | *A. niger* | Dry | Grass | MG659668 |
| ND75 | *A. flavus* | Dry | Mixed Ration | MG659669 |
| ND76 | *A. flavus* | Dry | Brewers’ Spent Grain | MG659670 |
| ND77 | *A. fumigatus* | Dry | Brewers’ Spent Grain | MG659671 |
| ND78 | *A. niger* | Dry | Mixed Ration | MG659672 |
| ND79 | *A. flavus* | Dry | Grass | MG659673 |
| ND80 | *A. niger* | Dry | Mixed Ration | MG659674 |
| ND81 | *A. fumigatus* | Dry | Grass | MG659675 |
| ND82 | *A. flavus* | Dry | Grass | MG659676 |
| ND83 | *A. niger* | Dry | Mixed Ration | MG659677 |
| ND84 | *A. tubingensis* | Dry | Concentrate | MG659678 |
| ND85 | *A. niger* | Dry | Mixed Ration | MG659679 |
| ND86 | *A. foetidus* | Dry | Grass | MG659680 |
| ND87 | *A. fumigatus* | Dry | Brewers’ Spent Grain | MG659681 |
| ND88 | *A. fumigatus* | Dry | Mixed Ration | MG659682 |
| ND89 | *A. niger* | Dry | Grass | MG659683 |
| ND90 | *A. flavus* | Dry | Grass | MG659684 |
| ND93 | *A. parasiticus* | Dry | Mixed Ration | MG659687 |
| ND94 | *A. tubingensis* | Dry | Grass | MG659688 |
| ND96 | *A. oryzae* | Dry | Grass | MG659690 |
| ND97 | *A. niger* | Dry | Brewers’ Spent Grain | MG659691 |
| ND98 | *A. flavus* | Dry | Grass | MG659692 |
| ND99 | *A. flavus* | Dry | Grass | MH270605 |
| ND100 | *A. chevalieri* | Dry | Mixed Ration | MH270606 |
| ND102 | *A. fumigatus* | Dry | Brewers’ Spent Grain | MH270608 |
| ND103 | *A. flavus* | Dry | Concentrate | MH270609 |
| ND104 | *A. sydowii* | Dry | Brewers’ Spent Grain | MH270610 |
| ND106 | *A. flavus* | Dry | Mixed Ration | MH270612 |
| ND107 | *A. brasiliensis* | Dry | Grass | MH270613 |
| ND109 | *A. flavus* | Dry | Concentrate | MH270615 |
| NR1 | *A. niger* | Rainy | Mixed Ration | MH270529 |
| NR2 | *A. ochraceus* | Rainy | Mixed Ration | MH270530 |
| NR3 | *A. flavus* | Rainy | Mixed Ration | MH270531 |
| NR6 | *A. fumigatus* | Rainy | Mixed Ration | MH270534 |
| NR7 | *A. fumigatus* | Rainy | Mixed Ration | MH270535 |
| NR10 | *A. flavus* | Rainy | Concentrate | MH270538 |
| NR11 | *A. welwitschiae* | Rainy | Concentrate | MH270539 |
| NR12 | *A. fumigatus* | Rainy | Concentrate | MH270540 |
| NR14 | *A. tubingensis* | Rainy | Concentrate | MH270542 |
| NR15 | *A. oryzae* | Rainy | Concentrate | MH270543 |
| NR16 | *A. flavus* | Rainy | Concentrate | MH270544 |
| NR17 | *A. fumigatus* | Rainy | Concentrate | MH270545 |
| NR18 | *A. fumigatus* | Rainy | Concentrate | MH270546 |
| NR20 | *A. flavus* | Rainy | Concentrate | MH270548 |
| NR22 | *A. phoenicis* | Rainy | Concentrate | MH270550 |
| NR24 | *A. niger* | Rainy | Mixed Ration | MH270552 |
| NR25 | *A. phoenicis* | Rainy | Mixed Ration | MH270553 |
| NR26 | *A. phoenicis* | Rainy | Concentrate | MH270554 |
| NR27 | *A. fumigatus* | Rainy | Mixed Ration | MH270555 |
| NR28 | *A. fumigatus* | Rainy | Mixed Ration | MH270556 |
| NR29 | *A. fumigatus* | Rainy | Concentrate | MH270557 |
| NR31 | *A. flavus* | Rainy | Brewers’ Spent Grain | MH270559 |
| NR32 | *A. fumigatus* | Rainy | Brewers’ Spent Grain | MH270560 |
| NR33 | *A. niger* | Rainy | Mixed Ration | MH270561 |
| NR34 | *A. awamori* | Rainy | Mixed Ration | MH270562 |
| NR35 | *A. oryzae* | Rainy | Mixed Ration | MH270563 |
| NR36 | *A. fumigatus* | Rainy | Mixed Ration | MH270564 |
| NR37 | *A. fumigatus* | Rainy | Mixed Ration | MH270565 |
| NR38 | *A. fumigatus* | Rainy | Mixed Ration | MH270566 |
| NR39 | *A. phoenicis* | Rainy | Mixed Ration | MH270567 |
| NR40 | *A. parvisclerotigenus* | Rainy | Mixed Ration | MH270568 |
| NR41 | *A. fumigatus* | Rainy | Grass | MH270569 |
| NR43 | *A. niger* | Rainy | Concentrate | MH270571 |
| NR44 | *A. fumigatus* | Rainy | Concentrate | MH270572 |
| NR46 | *A. flavus* | Rainy | Mixed Ration | MH270574 |
| NR47 | *A. fumigatus* | Rainy | Mixed Ration | MH270575 |
| NR49 | *A. fumigatus* | Rainy | Mixed Ration | MH270577 |
| NR50 | *A. flavus* | Rainy | Mixed Ration | MH270578 |
| NR51 | *A. fumigatus* | Rainy | Mixed Ration | MH270579 |
| ­­­­­­­­­­­­NR52 | *A. chevalieri* | Rainy | Mixed Ration | MH270580 |
| NR53 | *A. flavus* | Rainy | Mixed Ration | MH270581 |
| NR54 | *A. fumigatus* | Rainy | Concentrate | MH270582 |
| NR57 | *A. oryzae* | Rainy | Mixed Ration | MH270585 |
| NR58 | *A. niger* | Rainy | Mixed Ration | MH270586 |
| NR59 | *A. fumigatus* | Rainy | Mixed Ration | MH270587 |
| NR62 | *A. fumigatus* | Rainy | Mixed Ration | MH270590 |
| NR63 | *A. niger* | Rainy | Mixed Ration | MH270591 |
| NR65 | *A. fumigatus* | Rainy | Mixed Ration | MH270593 |
| NR66 | *A. oryzae* | Rainy | Mixed Ration | MH270594 |
| NR67 | *A. japonicus* | Rainy | Mixed Ration | MH270595 |
| NR68 | *A. fumigatus* | Rainy | Mixed Ration | MH270596 |
| NR69 | *A. niger* | Rainy | Grass | MH270597 |
| NR70 | *A. oryzae* | Rainy | Grass | MH270598 |
| NR71 | *A. fumigatus* | Rainy | Grass | MH270599 |
| NR72 | *A. nomius* | Rainy | Grass | MH270600 |
| NR73 | *A. fumigatus* | Rainy | Grass | MH270601 |
| NR74 | *A. fumigatus* | Rainy | Grass | MH270602 |
| NR75 | *A. fumigatus* | Rainy | Concentrate | MH270603 |
| NR76 | *A. fumigatus* | Rainy | Grass | MH270604 |
