# Supplementary material for: Biodiversity of Aflatoxigenic Aspergillus Species in Dairy Feeds in Bulawayo, Zimbabwe
Source: Front Microbiol. 2021 Jan 21;11:599605. doi: 10.3389/fmicb.2020.599605 (PMC7859627; doi:10.3389/fmicb.2020.599605)
Supplement: Supplementary file 2 [file Table_2.docx]

**Table S2.** Determination of aflatoxin (AF) production using plate assays

| **Isolate Voucher** | **Species** | **YES** | **NRDCA** | **β-NRDCA** | **AF production** |
| --- | --- | --- | --- | --- | --- |
|  |  | **Pink Color** | **Yellow Ring/Flourescence** | **Yellow Ring/Flourescence** |  |
| ND1 | *A. niger* | - | -/- | -/- | **-** |
| ND2 | *A. tubingensis* | + | +/- | +/- | **-** |
| ND3 | *A. awamori* | - | -/- | -/- | **-** |
| ND4 | *A. niger* | - | -/- | -/- | **-** |
| ND5 | *A. tubingensis* | - | -/- | -/- | **-** |
| ND6 | *A. awamori* | - | -/- | -/- | **-** |
| ND7 | *A. niger* | - | -/- | -/- | **-** |
| ND8 | *A. tubingensis* | - | -/- | -/- | **-** |
| ND9 | *A. tubingensis* | + | -/- | -/- | **-** |
| ND10 | *A. niger* | - | +/- | +/- | **-** |
| ND11 | *A. niger* | - | -/- | -/- | - |
| ND12 | *A. niger* | - | -/- | -/- | - |
| ND13 | *A. niger* | - | -/- | -/- | - |
| ND20 | *A. fumigatus* | - | -/- | -/- | - |
| ND21 | *A. fumigatus* | - | -/- | -/- | - |
| ND25 | *A. flavus* | + | +/- | +/- | **-** |
| ND26 | *A. flavus* | + | +/+ | +/+ | **+** |
| ND27 | *A. nomius* | + | +/+ | +/+ | **+** |
| ND28 | *A. flavus* | - | -/- | -/- | **-** |
| ND29 | *A. oryzae* | - | +/+ | +/+ | **+** |
| ND30 | *A. flavus* | + | +/+ | +/+ | **+** |
| ND31 | *A. flavus* | + | +/+ | +/+ | **+** |
| ND32 | *A. parasiticus* | + | +/+ | +/+ | **+** |
| ND33 | *A. flavus* | + | +/+ | +/+ | **+** |
| ND34 | *A. flavus* | + | +/+ | +/+ | **+** |
| ND35 | *A. oryzae* | + | +/+ | +/+ | **+** |
| ND36 | *A. flavus* | + | +/+ | +/- | **+** |
| ND37 | *A. flavus* | + | +/+ | +/+ | **+** |
| ND38 | *A. flavus* | + | +/+ | +/+ | **+** |
| ND39 | *A. oryzae* | + | +/+ | +/+ | **+** |
| ND40 | *A. flavus* | + | +/+ | +/+ | **+** |
| ND41 | *A. flavus* | + | +/+ | +/+ | **+** |
| ND44 | *A. niger* | - | -/- | -/- | - |
| ND45 | *A. cristatus* | - | -/- | -/- | - |
| ND51 | *A. flavus* | + | +/+ | +/+ | + |
| ND52 | *A. flavus* | + | +/+ | +/+ | + |
| ND54 | *A. awamori* | - | -/- | -/- | - |
| ND55 | *A. niger* | - | -/- | -/- | - |
| ND56 | *A. niger* | - | -/- | -/- | - |
| ND57 | *A. fumigatus* | - | -/- | -/- | - |
| ND58 | *A. niger* | - | -/- | -/- | - |
| ND59 | *A. flavus* | + | +/+ | +/+ | + |
| ND60 | *A. awamori* | - | -/- | -/- | - |
| ND61 | *A. fumigatus* | - | -/- | -/- | - |
| ND62 | *A. niger* | - | -/- | -/- | - |
| ND63 | *A. flavus* | + | +/- | +/- | - |
| ND64 | *A. niger* | - | -/- | -/- | - |
| ND65 | *A. niger* | - | -/- | -/- | - |
| ND67 | *A. niger* | - | -/- | -/- | - |
| ND68 | *A. niger* | - | -/- | -/- | - |
| ND69 | *A. fumigatus* | - | -/- | -/- | - |
| ND71 | *A. niger* | - | -/- | -/- | - |
| ND73 | *A. fumigatus* | - | -/- | -/- | - |
| ND74 | *A. niger* | - | -/- | -/- | - |
| ND75 | *A. flavus* | - | +/- | +/- | - |
| ND76 | *A. flavus* | - | +/- | +/- | - |
| ND77 | *A. fumigatus* | - | -/- | -/- | - |
| ND78 | *A. niger* | - | -/- | -/- | - |
| ND79 | *A. flavus* | + | +/+ | +/+ | + |
| ND80 | *A. niger* | - | -/- | -/- | - |
| ND81 | *A. fumigatus* | - | -/- | -/- | - |
| ND82 | *A. flavus* | - | -/- | -/- | - |
| ND83 | *A. niger* | - | -/- | -/- | - |
| ND84 | *A. tubingensis* | - | -/- | -/- | - |
| ND85 | *A. niger* | - | -/- | -/- | - |
| ND86 | *A. foetidus* | - | -/- | -/- | - |
| ND87 | *A. fumigatus* | - | -/- | -/- | - |
| ND88 | *A. fumigatus* | - | -/- | -/- | - |
| ND89 | *A. niger* | - | -/- | -/- | - |
| ND90 | *A. flavus* | - | -/+ | -/+ | - |
| ND93 | *A. parasiticus* | + | +/+ | +/+ | + |
| ND94 | *A. tubingensis* | - | -/- | -/- | - |
| ND96 | *A. oryzae* | + | +/+ | +/+ | + |
| ND97 | *A. niger* | - | -/- | -/- | - |
| ND98 | *A. flavus* | - | -/- | -/- | - |
| ND99 | *A. flavus* | + | +/+ | +/+ | + |
| ND100 | *A. chevalieri* | - | -/- | -/- | - |
| ND102 | *A. fumigatus* | - | -/- | -/- | - |
| ND103 | *A. flavus* | + | -/- | -/- | - |
| ND104 | *A. sydowii* | - | -/- | -/- | - |
| ND106 | *A. flavus* | + | +/- | +/- | - |
| ND107 | *A. brasiliensis* | - | -/- | -/- | - |
| ND109 | *A. flavus* | - | -/- | -/- | - |
| NR1 | *A. niger* | - | -/- | -/- | - |
| NR2 | *A. ochraceus* | + | +/+ | +/+ | + |
| NR3 | *A. flavus* | + | +/- | -/- | - |
| NR6 | *A. fumigatus* | - | -/- | -/- | - |
| NR7 | *A. fumigatus* | - | -/- | -/- | - |
| NR10 | *A. flavus* | + | +/- | -/- | - |
| NR11 | *A. welwitschiae* | - | -/- | -/- | - |
| NR12 | *A. fumigatus* | - | +/+ | -/+ | - |
| NR14 | *A. tubingensis* | - | -/- | -/- | - |
| NR15 | *A. oryzae* | - | -/- | -/- | - |
| NR16 | *A. flavus* | - | -/- | - /- | - |
| NR17 | *A. fumigatus* | - | -/- | -/+ | - |
| NR18 | *A. fumigatus* | - | -/- | -/- | - |
| NR20 | *A. flavus* | + | +/+ | +/+ | + |
| NR22 | *A. phoenicis* | - | -/- | -/- | - |
| NR24 | *A. niger* | - | -/- | -/- | - |
| NR25 | *A. phoenicis* | - | -/- | -/- | - |
| NR26 | *A. phoenicis* | - | -/- | -/- | - |
| NR27 | *A. fumigatus* | - | -/- | -/- | - |
| NR28 | *A. fumigatus* | - | -/+ | -/+ | - |
| NR29 | *A. fumigatus* | - | -/- | -/- | - |
| NR31 | *A. flavus* | + | +/+ | +/+ | + |
| NR32 | *A. fumigatus* | - | -/- | -/- | - |
| NR33 | *A. niger* | - | -/- | -/- | - |
| NR34 | *A. awamori* | - | -/- | -/- | - |
| NR35 | *A. oryzae* | + | +/- | - /- | - |
| NR36 | *A. fumigatus* | - | -/- | -/- | - |
| NR37 | *A. fumigatus* | + | +/+ | +/+ | + |
| NR38 | *As. fumigatus* | - | -/- | -/- | - |
| NR39 | *A. phoenicis* | - | -/- | -/- | - |
| NR40 | *A. parvisclerotigenus* | - | -/- | - /- | - |
| NR41 | *A. fumigatus* | - | -/- | -/- | - |
| NR43 | *A. niger* | - | -/- | -/- | - |
| NR44 | *A. fumigatus* | - | -/+ | -/+ | - |
| NR46 | *A. flavus* | + | +/+ | +/+ | + |
| NR47 | *A. fumigatus* | - | -/- | - /+ | - |
| NR49 | *A. fumigatus* | - | -/+ | -/+ | - |
| NR50 | *A. flavus* | - | -/- | -/- | - |
| NR51 | *A. fumigatus* | - | -/+ | -/+ | - |
| NR52 | *A. chevalieri* | - | -/- | -/- | - |
| NR53 | *A. flavus* | - | -/- | -/- | - |
| NR54 | *A. fumigatus* | - | -/+ | -/+ | - |
| NR57 | *A. oryzae* | + | +/+ | +/+ | + |
| NR58 | *A. niger* | + | +/- | +/- | - |
| NR59 | *A. fumigatus* | - | -/+ | -/+ | - |
| NR62 | *A. fumigatus* | - | -/+ | -/+ | - |
| NR63 | *A. niger* | - | -/- | -/- | - |
| NR65 | *A. fumigatus* | - | -/- | -/- | - |
| NR66 | *A. oryzae* | + | +/+ | +/+ | + |
| NR67 | *A. japonicus* | + | +/- | +/+ | - |
| NR68 | *A. fumigatus* | - | -/- | -/- | - |
| NR69 | *A. niger* | - | -/- | -/- | - |
| NR70 | *A. oryzae* | + | +/- | +/- | - |
| NR71 | *A. fumigatus* | - | -/- | -/- | - |
| NR72 | *A. nomius* | + | +/- | +/- | - |
| NR73 | *A. fumigatus* | - | -/- | -/- | - |
| NR74 | *A. fumigatus* | - | -/- | -/- | - |
| NR75 | *A. fumigatus* | - | -/- | -/- | - |
| NR76 | *A. fumigatus* | - | -/+ | -/+ | - |
