# Supplementary material for: Biodiversity of Aflatoxigenic Aspergillus Species in Dairy Feeds in Bulawayo, Zimbabwe
Source: Front Microbiol. 2021 Jan 21;11:599605. doi: 10.3389/fmicb.2020.599605 (PMC7859627; doi:10.3389/fmicb.2020.599605)
Supplement: Supplementary file 4 [file Table_4.docx]

**Table S4**. Determination of aflatoxigenic producing potential of isolates from section *Flavi*.

| **Isolate Voucher** | **YES** | **NRDCA** | **β-NRDCA** | **Aflatoxin cluster genes** | | | | | **Aflatoxin production** | **Species** | **GeneBank**  **Accession #** |
| --- | --- | --- | --- | --- | --- | --- | --- | --- | --- | --- | --- |
|  | **Pink color** | **Yellow Ring/Fluorescence** | **Yellow Ring/Fluorescence** | ***aflD*** | ***aflR*** | ***aflS*** | ***aflM*** | ***aflP*** |  |  |  |
| NR3 | + | +/- | - /- | + | + | + | + | + | **+** | *A. flavus* | MH270531 |
| NR10 | + | +/- | - /- | - | - | - | - | + | **-** | *A. flavus* | MH270538 |
| NR15 | - | -/- | -/- | - | - | - | - | + | - | *A. oryzae* | MH270543 |
| NR16 | - | -/- | -/- | + | - | - | - | - | **-** | *A. flavus* | MH270544 |
| NR20 | + | +/+ | +/+ | + | - | - | - | + | **-** | *A. flavus* | MH270548 |
| NR31 | + | +/+ | +/+ | + | + | + | - | + | **+** | *A. flavus* | MH270559 |
| NR35 | + | +/+ | - /- | + | + | + | - | + | **+** | *A. oryzae* | MH270563 |
| NR40 | - | -/- | - /- | + | + | + | + | + | **±** | *A. parvisclerotigenus* | MH270568 |
| NR46 | + | +/+ | +/+ | + | - | + | + | + | **+** | *A. flavus* | MH270574 |
| NR50 | - | -/- | -/- | + | + | + | + | + | **±** | *A. flavus* | MH270578 |
| NR53 | - | -/- | -/- | - | - | - | - | - | **-** | *A. flavus* | MH270581 |
| NR57 | + | +/+ | +/+ | + | - | - | - | - | **-** | *A. oryzae* | MH270585 |
| NR66 | + | +/+ | +/+ | + | + | - | + | + | **+** | *A. oryzae* | MH270594 |
| NR70 | + | +/- | +/+ | - | - | - | + | + | **-** | *A. oryzae* | MH270598 |
| NR72 | + | +/- | +/+ | + | - | - | + | + | **±** | *A. nomius* | MH270600 |
| ND25 | + | +/- | +/+ | - | - | - | - | - | **-** | *A. flavus* | MG659619 |
| ND26 | + | +/+ | +/+ | + | + | + | - | + | **+** | *A. flavus* | MG659620 |
| ND27 | + | +/+ | +/+ | + | + | + | + | + | **+** | *A. nomius* | MG659621 |
| ND28 | - | -/- | -/- | + | - | - | + | - | **-** | *A. flavus* | MG659622 |
| ND29 | + | +/+ | +/+ | + | + | + | - | + | **+** | *A. oryzae* | MG659623 |
| ND30 | + | +/+ | +/+ | + | + | + | - | + | **+** | *A. flavus* | MG659624 |
| ND31 | + | +/+ | +/+ | + | - | + | + | - | **+** | *A. flavus* | MG659625 |
| ND32 | + | +/+ | +/+ | + | - | - | - | + | **+** | *A. parasiticus* | MG659626 |
| ND33 | + | +/+ | +/+ | + | + | + | + | + | **+** | *A. flavus* | MG659627 |
| ND34 | + | +/+ | +/+ | + | + | + | - | + | **+** | *A. flavus* | MG659628 |
| ND35 | + | +/+ | +/+ | + | - | + | - | + | **+** | *A. oryzae* | MG659629 |
| ND36 | + | +/+ | +/- | - | - | - | - | + | **-** | *A. flavus* | MG659630 |
| ND37 | + | +/+ | +/+ | + | - | + | + | + | **+** | *A. flavus* | MG659631 |
| ND38 | + | +/+ | +/+ | + | + | + | + | - | **+** | *A. flavus* | MG659632 |
| ND39 | + | +/+ | +/+ | + | - | + | - | - | **+** | *A. oryzae* | MG659633 |
| ND40 | + | +/+ | +/+ | + | + | + | + | + | **+** | *A. flavus* | MG659634 |
| ND41 | + | +/+ | +/+ | + | + | + | + | + | **+** | *A. flavus* | MG659635 |
| ND51 | + | +/+ | +/+ | + | - | + | - | + | **+** | *A. flavus* | MG659645 |
| ND52 | + | +/+ | +/+ | + | - | + | - | - | **+** | *A. flavus* | MG659646 |
| ND59 | + | +/+ | +/+ | + | - | + | - | - | **+** | *A. flavus* | MG659653 |
| ND63 | + | +/- | +/- | - | - | - | - | - | **-** | *A. flavus* | MG659657 |
| ND75 | - | +/- | +/- | + | - | + | - | + | **-** | *A. flavus* | MG659669 |
| ND76 | - | +/- | +/- | + | - | - | - | + | **-** | *A. flavus* | MG659670 |
| ND79 | + | +/+ | +/+ | - | - | - | - | + | **-** | *A. flavus* | MG659673 |
| ND82 | - | -/- | -/- | - | + | + | + | + | **-** | *A. flavus* | MG659676 |
| ND90 | - | -/+ | -/+ | - | - | + | - | - | **-** | *A. flavus* | MG659684 |
| ND93 | + | +/+ | +/+ | - | + | + | - | - | **+** | *A. parasiticus* | MG659687 |
| ND96 | + | +/+ | +/+ | - | + | + | + | + | **+** | *A. oryzae* | MG659690 |
| ND98 | - | -/- | -/- | - | - | - | - | - | - | *A. flavus* | MG659692 |
| ND99 | + | +/+ | +/+ | + | + | + | + | + | **+** | *A. flavus* | MH270605 |
| ND103 | + | -/- | -/- | - | - | + | + | + | **-** | *A. flavus* | MH270609 |
| ND106 | + | +/+ | +/- | - | - | - | - | - | **-** | *A. flavus* | MH270612 |
| ND109 | - | -/- | -/- | - | - | - | - | - | **-** | *A. flavus* | MH270615 |

**Key**

**MR**= Mixed Ration, **CN=** Concentrate, **GR=** Grass, **BSG=** Brewers’ Spent Grain, +=present, - = absent, ± = potential producer
